# Unmet Needs in the Pathogenesis and Treatment of Systemic Lupus Erythematosus

**DOI:** 10.1007/s12016-017-8640-5

**Published:** 2017-08-29

**Authors:** Jyoti Bakshi, Beatriz Tejera Segura, Christopher Wincup, Anisur Rahman

**Affiliations:** Centre for Rheumatology Research, Rayne Institute, University College London, Room 412, Fourth Floor, 5 University Street WC1E 6JF, London, UK

**Keywords:** Systemic lupus erythematosus, Cardiovascular disease, Fatigue, Biologics, Clinical trials

## Abstract

Systemic lupus erythematosus (SLE) is an autoimmune rheumatic disease with a prevalence of approximately 1 in 1000. Over the last 30 years, advances in treatment such as use of corticosteroids and immunosuppressants have improved life expectancy and quality of life for patients with lupus and the key unmet needs have therefore changed. With the reduced mortality from disease activity, development of cardiovascular disease (CVD) has become an increasingly important cause of death in patients with SLE. The increased CVD risk in these patients is partly, but not fully explained by standard risk factors, and abnormalities in the immune response to lipids may play a role. Invariant natural killer T cells, which are triggered specifically by lipid antigens, may protect against progression of subclinical atherosclerosis. However, currently our recommendation is that clinicians should focus on optimal management of standard CVD risk factors such as smoking, blood pressure and lipid levels. Fatigue is one of the most common and most limiting symptoms suffered by patients with SLE. The cause of fatigue is multifactorial and disease activity does not explain this symptom. Consequently, therapies directed towards reducing inflammation and disease activity do not reliably reduce fatigue and new approaches are needed. Currently, we recommend asking about sleep pattern, optimising pain relief and excluding other causes of fatigue such as anaemia and metabolic disturbances. For the subgroup of patients whose disease activity is not fully controlled by standard treatment regimes, a range of different biologic agents have been proposed and subjected to clinical trials. Many of these trials have given disappointing results, though belimumab, which targets B lymphocytes, did meet its primary endpoint. New biologics targeting B cells, T cells or cytokines (especially interferon) are still going through trials raising the hope that novel therapies for patients with refractory SLE may be available soon.

## Introduction

Systemic lupus erythematosus (SLE) is an autoimmune rheumatic disease that affects women nine times more commonly than men and typically presents between the ages of 20 and 40. In the UK, it has an incidence of 4.91 per 100,000 per year and a prevalence of 97 per 100,000 [1]. There are two different sets of classification criteria for SLE: the American College of Rheumatology (ACR) criteria [2] and the Systemic Lupus International Collaborative Clinics criteria [3]. The SLICC criteria are slightly less specific and more sensitive than the ACR criteria but both recognise the principles that SLE is characterised by the presence of autoantibodies and can cause clinical effects in multiple different organs or tissues.

Mortality from SLE has improved dramatically over the last 50 years from a 5-year survival of 50% in the 1950s [4] to 95% now [5]. This improvement was primarily brought about by the introduction of treatment with corticosteroids and immunosuppression, first with cyclophosphamide [6] and azathioprine and later with mycophenolate [7]. As a result, there are fewer deaths from active lupus and a higher proportion of deaths occur from comorbidities such as cardiovascular disease and infection [8].

In parallel with these improvements, the major unmet needs for patients with lupus have changed over time. Thus, as these patients live longer, the development of premature cardiovascular disease has become an important issue. Symptom control remains important and fatigue is one of the most common, limiting and difficult-to-treat symptoms in patients with SLE. Finally, an important subgroup of patients with SLE is refractory to treatment with standard regimes of corticosteroids and immunosuppressants. Considerable effort has been directed towards development of new biologic agents for this refractory subgroup. In this review, we will concentrate on three major unmet needs in the management of patients with SLE, as follows:

1. Management of cardiovascular disease risk in patients with SLE
2. Management of fatigue in patients with SLE
3. Management of refractory SLE.

## Cardiovascular Disease in SLE

### Evidence for Increased Risk of CVD in SLE

Patients with SLE are at an increased risk of developing ischaemic heart disease or stroke—together termed cardiovascular disease (CVD)—compared to healthy people of the same age and gender. A large multinational study of 9547 patients with SLE [8] recorded 1255 deaths and 313 of these were due to CVD. The presence of SLE in women between the ages of 35 and 44 increases the risk of developing coronary disease by 50-fold, though across all ages the increased risk is less, at seven–tenfold [9].

As well as established CVD, it has also been shown in a number of different studies using a variety of imaging techniques (such as vascular ultrasound and electron beam tomography) that lupus patients also have a considerably higher prevalence of asymptomatic atherosclerotic plaque compared to healthy controls [10, 11].

Whilst traditional CVD risk factors may well account for some of this increased risk, they fail to explain all of it. Studies have shown that traditional CVD risk calculators based on the Framingham risk equations underestimate CVD risk in lupus [12, 13]. After adjusting for Framingham risk factors, it has been reported that patients still have a seven–tenfold increased risk of CVD [14].

To explain this unattributed risk, several authors have postulated that the immune dysfunction that characterises SLE may also promote development of atherosclerosis, leading to CVD [15]. It has been difficult to obtain clear proof that specific immunological factors can do this, but if such mechanisms can be identified, it could help in the development of biomarkers to predict CVD risk in patients with SLE and also in designing immunomodulatory therapies to reduce this risk.

### Possible Immunological Mechanisms Linking Lipids and CVD in SLE

Inflammation and lipid dysfunction are central to the pathogenesis of atherosclerosis. Inflammation plays a role in both atherosclerotic plaque formation and plaque rupture that occurs during an acute event [16].

SLE is characterised by lipid defects and chronic inflammation. Links between lipids and the pathogenesis of atherosclerosis in SLE are not fully understood and are likely to be multifactorial. Possible mechanisms include:

- Immune complex deposition which may stimulate the accumulation of cholesterol in atherosclerotic plaques [17].
- Dysfunctional pro-inflammatory high-density lipoprotein (HDL) cholesterol commonly present among SLE patients may accelerate low-density lipoprotein oxidation and atherosclerosis [18].
- Apolipoprotein A1 (ApoA1) is the main constituent of HDL. Anti-HDL and/or anti-apoA1 antibodies could interfere with the protective functions of HDL and could thus promote development of CVD. Anti-apoA1 antibodies have been associated with acute coronary syndromes in the general population [19] and patients with rheumatoid arthritis [20].

#### Anti-apoA1—a Possible Link Between Disease Activity and Development of Atherosclerosis in Patients with SLE

Anti-apoA1 antibodies are potentially relevant to pathogenesis of CVD in patients with SLE. Anti-HDL and/or anti-apoA1 antibodies could interfere with the protective functions of HDL and could thus promote development of CVD. The presence of anti-apoA1 and/or anti-HDL is associated with reduced activity of the antioxidant enzyme paraoxonase in SLE. This could cause increased oxidation of low-density LDL, which is a risk factor for the development of atheroma [21]. O’Neill et al. studied levels of anti-HDL, anti-apoA1 and anti-C-reactive protein (anti-CRP) in patients with SLE classified according to disease activity [22]. Raised anti-apoA1 levels were clearly associated with both current and persistent disease activity in both prospective and retrospective analyses and in longitudinal and cross-sectional studies [22]. However, in a subsequent study, the same group did not find any statistically significant association between levels of IgG anti-apoA1 and the development of atherosclerosis in patients with SLE [23].

#### Invariant Natural Killer Cells (iNKT Cells)

Invariant natural killer T cells (*i*NKT) are a small population of specialised immune cells that constitute less than 0.1% of peripheral blood mononuclear cells. They are thought to be important in a broad range of immune responses and are unique in that they respond to lipid antigens presented by the CD1d molecule on antigen presenting cells [24].

*i*NKT cells could potentially play a role in both autoimmunity and atherosclerosis. *i*NKT cells are reduced in number and have impaired function in SLE suggesting a protective role in autoimmunity [25]. Work from mouse models suggests a potential pro-atherogenic function of iNKT cells [26]; however, human studies have suggested that iNKT cells may have a protective role in the early stages of atherosclerosis by promoting atherosclerosis resolution via increased IL-10 production [27].

Smith et al. at University College London investigated the role of *i*NKT cells in the development of subclinical atherosclerosis in 100 patients with SLE who had no previous history of CVD, using vascular ultrasound to quantify the presence of plaque and its composition [28]. In this SLE patient cohort, 36% had subclinical plaque, supporting previous studies [11]. SLE patients with pre-clinical plaque (SLE-P) had a distinct anti-inflammatory *i*NKT cell profile characterised by increased activation and IL-4 production, which correlated with serum lipid expression levels and altered lipoprotein composition. *i*NKT cells differentiated in the presence of serum from SLE-P patients induced the polarisation of M2-like macrophages (anti-inflammatory*)* in vitro. This protective *i*NKT cell phenotype was lost in SLE patients who had a cardiovascular event. These SLE-CVD patients had a different *i*NKT cell phenotype, characterised by low peripheral blood frequency, increased CD8^+^ phenotype and a low expression of IL-4 and IFN-ɣ compared to SLE-P and SLE-NP patients. This was associated with increased expression of pro-inflammatory monocytes (CD14 and CD16^+^) and reduced M2-like monocyte frequency. The results support an athero-protective role for *i*NKT cells driven by serum lipids during the early stages of atherosclerosis, which was lost or overwhelmed during the development of clinical atherosclerosis [28].

Smith et al. [28] also tested to see whether dyslipidaemia, a risk factor for atherosclerosis, was associated with the altered *i*NKT cell activation in SLE-P patients. Serum lipid composition analysed using proton nuclear magnetic resonance spectroscopy revealed elevated concentrations of VLDL but not LDL or HDL particles in SLE-P compared to SLE-NP patients. The proportions of free cholesterol, cholesterol esters, phospholipids and triglycerides carried by lipoprotein subclasses (particularly in VLDL lipoproteins) as well as other serum metabolomic components were significantly different in SLE-P compared to SLE-NP patients. In comparison, serum lipid measures quantified in the clinical laboratory as part of routine patient management were within normal clinical ranges [28].

In conclusion, the results published suggest that *i*NKT cells may have a unique phenotype during different stages of atherosclerosis and that this may be driven by dyslipidaemias.

It may well be that the traditional targets such as LDL cholesterol targeted by statins are in fact not the correct lipids that should be targeted in lupus and that other serum lipids such as VLDL may be more relevant for patients with SLE [29].

### How Should We Manage CVD Risk in SLE?

In the future, identification of immunological factors may enable us to identify a subgroup of patients who are at a higher risk of CVD to enable risk stratification and more stringent control of disease activity and traditional CVD risk factors.

At present, however, management of traditional risk factors is the best strategy for modifying CVD risk. SLE subjects should be screened annually for cardiac risk factors—one report showed that only 26% of patients had four cardiac risk factors assessed annually [30]. In the Systemic Lupus International Collaborative Clinics cohort, hypercholesterolemia was not treated in up to two thirds of patients [30].

Management of CVD risk can be undertaken by a target-based approach. As SLE is a very high risk condition for CVD, some experts have suggested that it can be viewed as a “coronary heart disease equivalent” like diabetes and therefore targets for hypertension and raised cholesterol should be adjusted accordingly [31]. Table 1 outlines a possible scheme for targeting these risk factors in patients with SLE.

**Table 1:** Possible scheme for assessment of traditional CVD risk factors in SLE

Risk factor	Prevalence in SLE	Frequency of assessment	Target and treatment options
Smoking	17–21% of patients with SLE smoke	Annually	Stop smoking (in all patients) Referral to cessation clinics, specialist nurse, drug treatments e.g. nicotine replacement therapy
Cholesterol	Hypercholesterolemia 34–51% Typically low HDL and raised triglycerides (from inflammatory disease ± steroids)	Annual fasting lipids namely total, HDL, LDL, and triglycerides	Adult Treatment Panel 111 guidelines [22]—LDL cholesterol key lipid parameter: 1. LDL cholesterol < 2.6 mmol/l no action review annually 2. LDL 2.6–3.4 mmol/l therapeutic lifestyle changes- diet and weight reduction 3. LDL > 3.4 mmol/l or still > 2.6 despite lifestyle modification— drug therapy, e.g. statin
Body mass index	Frequently seen in truncal distribution in SLE	Annually	>25 kg/m^2^ (over weight): Consider steroid dose adjustment, lifestyle modification, exercise programmes, dietary education, reducing alcohol. Monitor over set period of time. If above fail, then drug treatment as recommended by NICE [22]
Diabetes mellitus	5–7%	Screening at every visit as part of SLE assessment with a urine sample Random glucose—annually at least Monitor those on high dose steroids more closely	DM diagnosed in presence of fasting glucose ≥ 7 mmol/l or random blood glucose or ≥ 11 mmol/l Referral to specialist in diabetes for all patients with diabetes.
Blood pressure	Common in SLE	At every clinic visit and at least annually	1. Ideal target < 130/80 mmHg 2. > 140/90 review closely and lifestyle measures offered. Steroid treatment reviewed and renal function monitored 3. If > 140/90 despite measures in two-drug treatment based on age and comorbidities. Start ACE inhibitors in all with renal lupus 4. Review BP every 3 months after starting treatment and ideally be kept < 130/80

Anti-malarials are commonly used in SLE and have been found to have cardioprotective properties with decreased prevalence of carotid plaque [32] reduced vascular stiffness [33] and cholesterol lowering effects on patients in patients on corticosteroids. In a nested case-control study, hydroxychloroquine demonstrated a risk reduction of 68% for all thromboembolic events [34].

Mycophenolate mofetil has shown favourable results in mouse models with atherosclerosis [35]**.** In non-SLE patients, short courses of mycophenolate have shown decreased T cell activation in carotid plaques. Although studies in SLE patients are yet to show favourable outcomes [36], larger studies are required.

Potential novel therapies include pathways involved in atherosclerosis and endothelial dysfunction. Type 1 interferons are an example of such mediators and could potentially be targeted to reduce CVD risk in SLE (see section on refractory SLE below).

## Fatigue in Systemic Lupus Erythematosus

The majority of patients with lupus often report fatigue to be the single most troublesome symptom of their illness that negatively impacts on their quality of life [37] Between 80 and 90% of patients with lupus report fatigue to be the most debilitating symptom of their disease [38, 39]. Interestingly, patients with both clinically and serologically well-controlled lupus continue to demonstrate marked levels of fatigue, in spite of apparent disease remission [40–42]. This troublesome symptom has wide ranging effects on quality of life, resulting in significant disruption to daily function [43]. This has wider implications for work, exercise, and leisure activities with a high prevalence of employment disability reported [44]. Furthermore, fatigue is associated with impaired concentration, which often has implications on education, in particular in younger patients with lupus.

### Metabolic Disorders

In some cases, an obvious cause for fatigue is easily identifiable: for example, intercurrent metabolic disorders, such as hypothyroidism, which should be screened for. In addition, anaemia is the most common haematological manifestation of lupus and can frequently contribute to fatigue [45]. The most common forms of anaemia in lupus are anaemia of chronic disease, iron deficiency anaemia and autoimmune haemolytic anaemia [45]. However, in the vast majority of cases, an identifiable cause for fatigue is not obviously detectable. A variety of factors have been proposed to play a role in the development of fatigue in lupus, although there is no widely accepted unifying mechanism described. It is likely that the aetiology of fatigue in lupus is multifactorial.

### Sleep Disturbance

One important factor to consider when assessing patients with high levels of fatigue is the role of sleep disturbance. This can include difficulty initiating sleep, maintaining sleep and early morning wakening. Problems with sleep have been widely reported in a number of autoimmune rheumatic conditions [46–48]. In a study of 81 female patients with lupus, Palagini et al. reported insomnia and poor sleep quality was common. Nearly two thirds of patients described poor sleep quality and/or early morning awakening (of which one third also reported insomnia) [49]. A key predictor of poor sleep quality in patients with lupus is pain [50]. It has been previously found that fatigue is closely associated with patient pain scores [51]. In a study by Gudbjornsson and Hetta, patients with lupus were found to report more pain when trying to fall asleep and during the night when compared with healthy controls [52]. Joint pain from arthralgia and arthritis is commonly seen in lupus and this can cause significant sleep disruption. Fibromyalgia, a condition that commonly co-exists with lupus, is another important factor to consider as this can result in nocturnal pain contributing to sleep disruption and fatigue [53, 54]. Other factors that lead to sleep disruption in lupus patients include respiratory symptoms and restless leg syndrome [55, 56]. Intercurrent depression and anxiety can also negatively impact on sleep. Da Costa et al. reported that in a sample of 100 women with lupus, depressed mood and a lack of physical activity significantly contributed to poor sleep quality [57]. The impact of sleep disturbance leads to significant reduction in physical activity, which has a variety of negative effects on psychological well-being [58]. A number of studies have highlighted high levels of depression and anxiety in patients with lupus [59, 60]. The presence of these mental health problems is commonly associated with fatigue in lupus patients [38, 47]. Physical deconditioning is believed to also play a role in fatigue in lupus. Various studies have reported that patients with lupus have a lower cardiovascular capacity and reduced muscle strength when compared with the healthy population [38, 61, 62].

### Medication

Another important consideration in the development of fatigue in patients with lupus is the role of medication. Corticosteroids are well known to cause sleep disturbance that can cause or exacerbate fatigue [63]. A study by Jump et al. interestingly noted that patients taking non-steroidal anti-inflammatory drugs (NSAIDs) were found to have higher levels of fatigue when compared with those who did not [64]. However, this was noted to be no longer significant when controlled for pain scores [64]. In addition, opiate-based analgesia, anti-depressants and anxiolytics can commonly have sedating side effects, which can exacerbate fatigue [65].

### Central Nervous System Involvement in Lupus

Lupus is known to cause significant and sometimes severe neurological involvement resulting in a number of diverse symptoms [66]. The various neurological and psychiatric manifestations that occur in lupus pose both diagnostic and therapeutic challenges. Lupus patients with central nervous system (CNS) involvement have been reported to have significant reduction in quality of life outcome measures when compared with those who have other serious manifestations of the disease (for example, renal involvement). Furthermore, patients with CNS involvement had significantly higher fatigue scores when compared with those who did not have CNS involvement (based on the SF-36 questionnaire) [67].

In some cases, in spite of no obvious cause and in the absence of apparently obvious disease activity, patients continue to report high levels of fatigue. A number of suggestions have been made including the possibility of subclinical central nervous system involvement, which may lead to fatigue. One possible explanation is that disruption and dysregulation within the blood-brain barrier (BBB) leaves the central nervous system open to the deleterious effects of infiltrating pro-inflammatory cytokines. This hypothesis is difficult to test due to obvious difficulties in monitoring CNS cytokines without invasive tests. In a recent study by Gulati et al., diffusion-weighted magnetic resonance imaging (MRI) was used to measure regional BBB integrity in patients with juvenile-onset lupus. It was noted that patients with lupus demonstrated increased regional capillary permeability within the BBB when compared with healthy controls [68]. How this relates to fatigue is yet to be fully established however.

In addition to disruption of the BBB, diffusion-weighted MRI has been used to assess for evidence of cortical damage in lupus. Wiseman et al. recently reported an increased presence of microstructural white matter damage in patients with lupus when compared with healthy controls of the same age [69]. However, in this study, there was no clear association between the prevalence of these white matter changes and the levels of fatigue seen. Interestingly, the study did note that lupus patients with poorer cognitive function had higher circulating levels of interleukin-6 (IL-6). The way in which IL-6 may result in the white matter changes and cognitive function is however not understood [69].

### Pro-inflammatory Cytokines and Potential Biomarkers for Fatigue

As mentioned, fatigue remains a troublesome symptom in patients with lupus who appear to have both clinically and serologically well-controlled disease [40]. Furthermore, there is no convincing evidence that fatigue levels are related to lupus disease activity [41]. In view of this, there has been an increased focus of late to identify surrogate biomarkers of fatigue in lupus. A number of serum markers have been investigated by means of investigating the potential pathogenesis and treatment of fatigue in lupus. Omdal et al. have focused on pro-inflammatory serum cytokines including TNF-α, IL-2, IL-6, IL-10, TGFβ- and interferon-α. However, there was no demonstrated correlation between serum concentrations of these circulating cytokines and levels of fatigue [41]. In addition, antiphospholipid antibodies (present in 30–40% of lupus patients [70]) were also measured, although similarly there was no significant association with fatigue in this case.

Abnormal oxidative metabolism and mitochondrial dysfunction have been proposed as possible mechanisms for fatigue in lupus. Reactive oxygen species (ROS) are produced as a result of normal aerobic mechanism. The clearance of these potentially damaging ROS is well maintained by several enzyme systems. Prolonged exposure to ROS is believed to result in oxidative damage to DNA [71] and is believed to have a role in the pathogenesis of lupus [72–74]. Segal et al. found that plasma levels of F_2_-isoprostane (a marker of oxidative stress) were increased in lupus patients with low disease activity when compared with healthy controls. Interestingly, levels of F2-isoprostane were a predictor for high fatigue scores measured by Fatigue Severity Scale (FSS). It was proposed that this may be a useful biomarker of mitochondrial function in lupus patients with high levels of fatigue [75].

### Treatment of Fatigue in Patients with SLE

As has already been outlined, there is no reliable treatment for fatigue in patients with lupus. Fatigue and quality of life outcomes are often measured in clinical trials in lupus. The BLISS trial investigated the use of belimumab (a B lymphocyte stimulator-specific inhibitor) in lupus. This trial reported an improvement in fatigue (measured by FACIT Fatigue score) as a secondary outcome [76].

Vitamin D deficiency is a common finding in both the general population and in patients with lupus [77, 78]. Previously, low level of vitamin D (25-hydroxyvitamin D3) has been found to correlate with increased lupus disease activity (although did not correlate with complement levels or damage scores) [79]. A recent randomised, double-blind placebo-controlled trial of weekly high dose (50,000 I/U) oral vitamin D supplementation in patients with juvenile-onset lupus was noted to show a significant improvement in terms of both disease activity (measured by SLEDAI) and fatigue levels (measured by the Kids Fatigue Severity Scale), which suggests that the treatment of low vitamin D levels may be useful in the management of patients with lupus [80].

Non-pharmacological treatment of fatigue in lupus has previously been investigated by Tench et al., in a trial that compared graded exercise, relaxation therapy and no intervention [81]. The exercise group was asked to complete three 30–50 min exercise sessions a week for a period of 12 weeks. Exercise predominantly included walking, cycling and swimming. By comparison, the group assigned to relaxation therapy was asked to listen to a 30-min relaxation tape three times a week. The final group was asked to continue with their usual daily routines. Fatigue levels were noted to significantly improve in the patient group undergoing graded exercise when compared with the other two groups at 12 weeks. However, these improvements were not maintained at 3-month follow-up. This was believed to be due to the fact that not all patients continued with the regular exercise regime [81].

Overall, recommendations for management of fatigue in patients with SLE are summarised in Table 2.

**Table 2:** Recommendations for the management of fatigue in lupus

1. Enquire about sleep disturbance (this usually has a bidirectional role in fatigue)
2. Address regular medication and reduce steroids to the minimal effective dose
3. Ensure adequate pain control
4. Screen for other exacerbating syndromes (such as fibromyalgia, depression and anxiety)
5. Identify any coexisting metabolic disorders (such as anaemia, hypothyroidism and vitamin D deficiency)
6. Encourage regular paced activity

In conclusion, fatigue is a prevalent problem faced by many patients with lupus. This is often independent of disease activity and remains problematic even in those with well-controlled disease. Escalation of standard immunosuppressive therapy is often ineffective in the treatment of fatigue. The causes of fatigue in lupus are poorly understood and likely to be multifactorial.

## Treatment of Refractory Lupus

This part of the review will focus on the pharmacological aspect of managing SLE patients who do not respond to conventional treatment such as current immunosuppressants and steroids.

### Steroids and Current Immunosuppressive Treatments

The use of corticosteroids revolutionised the treatment of SLE in the 1950s and still remains as the mainstay of treatment (Table 3). Nonetheless, they are not exempt from short-term and long-term side effects, which are major issues in SLE management. The risk of damage is higher with high doses albeit there is no safe dose for chronic use. It is well known that even small doses taken for a long period of time will increase the morbidity. In fact, there are studies which have shown that the hazard ratio for organ damage increases dramatically with prednisone doses of 6 to 12 mg/day, after adjusting for confounding factors by indication due to SLE disease activity [82]. In addition, development of osteopenia and osteoporosis is an important potential adverse effect of taking corticosteroids for long periods. Every attempt should be made to minimise the dose and duration of corticosteroid exposure [83].

**Table 3:** Current and future treatments for SLE

	Main indications	Potential risks (most common—but not an exhaustive list)
Current treatments for SLE		
Corticosteroids	• Acute flares of the disease • To maintain remission in patients with previous severe forms of lupus(e.g. renal/cerebral)	• Increased cardiovascular risk • Osteoporosis- Increased risk of fragility fractures • Cataracts • Avascular necrosis
Hydroxychloroquine chloroquine	• Mild SLE and SLE with organ involvement in combination with other immunosuppressants • In general, anti-malarials should be considered in every patient with SLE unless there are contra-indications	• Retinal toxicity • Gastrointestinal side effects
Azathioprine	• Maintenance therapy in cases with major organ involvement usually in combination with corticosteroids • Steroid-sparing agent in some cases	• Gastrointestinal side effects • Bone marrow toxicity (dose-dependent) • Infections • Hepatotoxicity
Mycophenolate mofetil	• Lupus nephritis (induction and maintenance therapy) • Same indications as azathioprine	• Gastrointestinal side effects • Bone marrow aplasia (very unlikely) • Opportunistic infections • Contra-indicated in pregnancy
Cyclophosphamide (usually intravenous—the low dose Eurolupus regime is often used)	• Lupus nephritis • CNS involvement—vasculitis • Alveolar haemorrhage	• Haemorrhagic cystitis • Bone marrow suppression • Opportunistic infections • Increased risk of cancer • Infertility
Methotrexate	• Joint involvement • Skin involvement • Steroid sparing agent in some cases	• Bone marrow suppression • Hepatotoxicity • Lung toxicity-lung fibrosis
Belimumab	• Skin involvement • Joint involvement • Limited evidence in major organ involvement • Elevated anti-dsDNA, low complement	• Increased risk of infections • Infusion reaction
Rituximab	• Lupus nephritis • Refractory thrombocytopenia or haemolytic anaemia • CNS involvement (case-report series)	• Increased risk of infections • Bone marrow suppression • Multifocal leukoencephalopathy • Infusion reaction
Future treatments for SLE		
Abatacept	• Lupus arthritis	• Increased risk of infections Ongoing clinical trial phase III
Fostamatinib (R788)	• Lupus nephritis	• Preliminary results in murine models • No data in humans
Laquinimod/paquinimod	• Lupus arthritis • Lupus nephritis	• Arthralgia • Myalgia • Pharyngolaryngeal pain • Dose-dependent side effects
Forigerimod (Lupuzor)	• Moderate active SLE	• Ongoing phase III trial
Sirukumab	• Skin disease • Mild SLE	• Reduction in white cell, neutrophil and platelet counts (dose-independent)
Sifalimumab	• Moderate active SLE	• Herpes zoster infections
Anifrolumab	• Moderate active SLE	• Ongoing phase III trial

Initially, cyclophosphamide was considered the standard of care in those patients with refractory lupus, particularly those with lupus nephritis. Nevertheless, it was associated with significant side effects including infections, malignancy and infertility. Therefore, lower doses of cyclophosphamide and/or replacement by mycophenolate became effective options for induction therapy and likewise, better safety treatments [84, 85]. Ethnicity seems to be important in the responses to these treatments. Previous studies have demonstrated that Afro-Caribbean patients have better response to mycophenolate than Caucasian and Asian patients [86].

Azathioprine and mycophenolate have been demonstrated to be effective options for maintenance therapy in randomised trials^.^ In the ALMS trial, mycophenolate was superior to azathioprine [87]; however, this did not remain true in the MAINTAIN trial [88].

Despite the dramatic improvements in the management of SLE brought about by use of these drugs, there are still many patients who fail to respond to those treatments. Novel approaches have been tried in order to assess these issues. For instance, calcineurin inhibitors such as ciclosporin and tacrolimus have shown hopeful results in SLE patients who did not respond to first-line therapies [89]. Therefore, it is imperative to develop large controlled clinical trials in order to confirm these prior findings.

### Biologic Therapy for SLE

Over the past decade, there has been a significant development in biologic therapies for SLE patients. These therapies can be divided into those directed at B cells and non-B cell targets. Moreover, T cell interactions or cytokines such as type I interferons are also emerging targets with pivotal roles in SLE pathogenesis.

### B cells as a Target for Treatment in SLE

#### Rituximab

B cells play a central role in the pathogenesis of lupus. Polyclonal B cell activation results in the production of autoantibodies directed against many nuclear, cytoplasmic and plasma membrane antigens. Almost 95% of patients with SLE have anti-nuclear antibodies (ANA). Many of the clinical manifestations of lupus are a result of immune complex deposition in tissues and subsequent multi-organ damage. Immune complexes are formed as anti-nuclear antibodies bind to nuclear material. B cells also perpetuate the inflammatory response by presenting auto-antigens to T cells and producing pro-inflammatory cytokines [90].

Rituximab, a chimeric anti-CD20 antibody, efficiently and reliably depletes CD20-positive B cells. Two large phase III randomised controlled trials involving patients with moderate to severe lupus but non-renal SLE (EXPLORER) [91] and lupus nephritis class III or IV (LUNAR) [92] have evaluated rituximab for treatment of SLE, although both failed to meet their primary endpoints of significant reduction of disease activity compared to placebo. In the EXPLORER trial, the group treated with rituximab had significant improvements in serology-reduced anti-dsDNA antibody (*p* = 0.006), increased C3 (*p* = 0.0029) and C4 levels (*p* = 0.0045)—at the termination of the trial, although there were no significant clinical improvements [91]. In the LUNAR trial, there was a trend to an improved response rate in Afro-Caribbean subjects treated with rituximab. A statistically significant serological improvement in the treatment group was found with anti-dsDNA antibodies falling (*p* = 0.007) and C3 levels rising (*p* = 0.03) [92].

A prospective observational study of rituximab [93] as part of corticosteroid-sparing regimen in lupus nephritis patients showed promising results with good renal outcomes in 45/50 patients treated on an open-label basis. This led to a multicentre randomised controlled trial (RITUXILUP) with rituximab as induction therapy followed by maintenance mycophenolate mofetil. However, that trial was recently halted due to difficulties with recruitment. The RING study (Rituximab for lupus Nephritis with Remission as a Goal) is another ongoing trial of rituximab which is an open-label, multicentre trial aiming to determine the efficacy of rituximab in achieving complete renal remission in lupus nephritis patients with persistent proteinuria. Patients who have been treated for a minimum of 6 months of standard immunosuppression may participate in the trial [ClinicalTrials.gov identifier:NCT01673295].

##### Belimumab

Belimumab is a fully humanised monoclonal antibody that binds to soluble human B lymphocyte stimulator (BLyS) and was the first new drug for SLE approved by the US authorities in 50 years. It has been studied in two large randomised controlled trials in SLE (BLISS-52 [*n* = 865] [76] and BLISS-76 [*n* = 819] [94]). These trials were performed in patients with active disease (defined by a SLE Disease Activity Score Index of ≥ 6**)** and the majority of patients entered with active arthritis and/or a rash. Patients were randomised to receive placebo plus or intravenous infusions of belimumab at 1 or 10 mg/kg at 2 and 4 weeks, and then every month until 48 weeks. Both of these studies excluded SLE patients with active lupus nephritis or neuropsychiatric disease. This exclusion is commonly seen in lupus trials and is important as those manifestations are often seen in patients with refractory SLE.

The belimumab studies, unlike those with rituximab, met their primary endpoints, with significantly greater response rates in the treatment groups than the placebo groups. Belimumab was more effective in SLE patients who were serologically active, that is those who were anti-dsDNA antibody positive and were hypocomplementemic [95]. The BLISS-LN phase III study has been devised to evaluate the efficacy and safety of belimumab plus standard of care in active lupus nephritis, and is recruiting patients [ClinicalTrials.gov identifier:NCT01639339].

#### Epratuzumab

Epratuzumab is a monoclonal antibody targeting CD22 receptors (a cell surface marker on transitional B cells and naïve mature B cells) leading to moderate B cell depletion via antibody-dependent cellular cytotoxicity (ADCC). There have been three different randomised controlled trials in moderate-to-severe active SLE. A phase II study (EMBLEM) was promising as a dose of 2400 mg per month in divided doses was safe and appeared to show improved efficacy compared to placebo [96]. However, two phase III studies failed to meet their primary endpoint and did not show benefit of epratuzumab over placebo plus standard therapy [97].

#### Atacicept

Atacicept is a recombinant human fusion protein that targets the BlyS/APRIL axis. There have been three phase II/III studies. One of these randomised 461 patients to placebo or two different doses of atacicept. The outcome measure was the ability to prevent flares of lupus in patients whose disease was inactive at the start of the trial period. As with other B cell targeting agents, atacicept led to an improvement in serology (anti-dsDNA and complement) but response was only better than placebo in the higher dose group. The trial in this higher dose group was halted due to two fatal infections [98].

### T cells as a Target for Treatment in SLE

T lymphocytes play a fundamental role in SLE. They enhance autoantibody production by B cells and are believed to trigger SLE disease. On the other hand, T cells themselves can only be stimulated by interaction with specialised antigen presenting cells (APCs), which include B cells. In an APC-T cell interaction, there are two points of contact between the cells. Firstly, the APC presents the antigen to the T cell in association with a major histocompatibility complex (MHC) molecule; this is the antigen-dependent interaction. Secondly, there is a co-stimulatory signal between pairs of ligands, one on the APC and one on the T cell, leading to the antigen-independent interaction [99].

Many drugs, which act as inhibitors of T cell/APC co-stimulation, have failed in different clinical trials. Some examples are anti-CD40L treatment such as IDEC-131 or BG9588, both humanised anti-CD40L antibodies. The first one failed in a phase II double-blind trial due to inefficacy over placebo [100]. The second one was associated with unacceptably high rate of thromboembolic events [101].

Abatacept is a fusion protein comprised of Fc portion of human IgG1 associated with a cytotoxic T lymphocyte antigen (CTLA4). It is a selective co-stimulation modulator as it inhibits the co-stimulation of T cells. A phase II trial of abatacept in non-renal SLE patients failed to meet its primary endpoint of reduction in new flares. A phase III trial of abatacept in proliferative lupus nephritis (classes III and IV) failed to meet its study endpoint of complete renal response [102]. However, a very strict endpoint definition of complete renal response was used in this study [102].

Considering the possible errors in the definitions of the outcomes, a re-analysis of the same study data was undertaken using outcome measures from the LUNAR and ALMS trials and did show a positive outcome in favour of abatacept therapy [103].

Other T cell targeting therapies are at a preliminary stage of development in the treatment of SLE. Tyrosine kinases play a central role in the signalling processes involved in the pathogenesis of autoimmune disease [104]. Spleen tyrosine kinase (Syk) is a cytoplasmic protein that is widely expressed in many cells of the immune system including B and T cells. Fostamatinib (R788), a Syk inhibitor has been studied in lupus-prone mice and it significantly prolonged survival delaying the onset of proteinuria and reducing kidney infiltrates. R788 has been studied in patients with rheumatoid arthritis (RA), but there have been no trials in SLE [104]. Other tyrosine kinase inhibitors including the JAK inhibitor, tofacitinib, which has been approved for RA, and baracitinib studied in RA, have not as yet been evaluated in SLE.

Several other T cell-targeting therapies at early stages of development in SLE include the immunomodulatory molecules such as the quinoline-3-carboxamide derivatives (laquinimod and paquinimod), U1 small ribonucleotide fragments (forigerimod) and sphingosine-1-phosphatase analogues (fingolimod and KRP-203). Laquinimod, developed for the treatment of multiple sclerosis, seems to decrease the activation of T cells via toll-like receptor 4, contributing to an anti-inflammatory effect. Two phase II randomised trials of laquinimod have been completed in lupus arthritis and lupus nephritis. Forigerimod (Lupuzor), which is a tolerogenic peptide, has been studied in a phase II trial encompassed 149 SLE patients showing some clinical benefit. It was well tolerated and efficacious with regard to SRI [105].

### Anti-interleukin-6 Therapies

IL-6 levels are found to be raised in the serum of SLE patients and it has both pro-inflammatory and anti-inflammatory effects.

Tocilizumab is a humanised monoclonal antibody targeted against the IL-6 receptor. A phase I trial of this biological treatment showed good tolerance to the drug with reduction in active urinary sediment and autoantibody titres [106]. In the study of Shirota et al., tocilizumab was used in 15 SLE patients with mild-moderate disease activity with a reduction in the activity of T cells and B cells [107]. Nonetheless, concerns were raised about its safety. There were dosage-related decreases in the absolute neutrophil count and clinically relevant infections; therefore, further studies are needed to define the role of tocilizumab in the SLE management.

Sirukumab is another monoclonal antibody against IL-6. Both safety and pharmacokinetics were evaluated in a phase I, double-blind, placebo-controlled study in patients with cutaneous or systemic lupus with mild, stable, active disease. The study demonstrated good tolerability of sirukumab, although it was associated with dose-independent reduction in white cell, absolute neutrophil and platelet counts [108]. A phase II randomised trial of CNTO 136 in patients with active lupus nephritis is completed and the results are awaited.

### Anti-tumour Necrosis Factor Therapies

The role of tumour necrosis factor (TNF)-α in SLE is controversial [109]. In different strains of lupus mice, the expression of TNF-α is often variable, and beneficial effects on the disease can be observed either after administration of TNF or upon blockade. In kidney inflammation, the renal expression of TNF is usually increased. In lupus mice, skin disease may be TNF-dependent, and anti-TNF treatment can cause worsening of nephritis. In humans with SLE, some studies have found relatively low concentrations of serum TNF-α, whilst other studies have observed elevated amounts or no significant differences between SLE patients and healthy controls. It is important to bear in mind that there is a balance between TNF and its soluble inhibitors (reviewed in [110]).

As it has been reported previously, the TNF blockers that have been successful in the management of RA, Crohn’s disease and psoriasis are known to induce autoantibodies and lupus-like syndromes. Therefore, their use in SLE is unclear and they are mainly reserved for cases where arthritis is a major feature. In small studies in SLE, anti-TNF therapy has been associated with improvement in polyarthritis, and nephritis [111, 112], but also with severe infusion reaction.

### Anti-interferon-α Therapies

Numerous studies have demonstrated the role of IFN-α in the pathogenesis of SLE. Murine studies with the New Zealand Black mouse model of SLE showed that genetic deficiency of the type I IFN receptor led to significantly reduced disease activity [113]. Moreover, serum interferon-α levels have been shown to correlate with disease activity and severity in SLE patients. Gene expression microarray profiles demonstrate upregulation of interferon-inducible genes [114], particularly in those with severe SLE manifestations such as lupus nephritis and neuropsychiatric disease. Sifalimumab is a fully human anti-interferon-α monoclonal antibody which was shown in a phase I study to induce a dose-dependent inhibition of type I interferon-induced mRNAs in patients with moderately active SLE. A phase IIb trial evaluating the efficacy and safety of sifalimumab in SLE achieved its primary endpoints using the SRI(4) and the more stringent SRI (6–8) [115].

Anifrolumab, which is an anti-interferon-α receptor 1 antibody, seemed to have more significant and sustained impact on the interferon gene signature than sifalimumab, taking into account the results of two phase II randomised open-label studies. A recent report of a phase IIb study showed that anifrolumab reduced disease activity more than placebo, particularly in patients with high interferon signature [116]. Therefore, anifrolumab has been selected for phase III studies.

Vaccination against IFN-α using IFN-α kinoid is another therapeutic approach that has been investigated in murine studies. The IFN-α immunogen induced transient neutralising antibodies to the cytokine without apparent side effects, and the lupus manifestations were delayed or prevented in kinoid-vaccinated mice. These studies suggest a new therapeutic strategy for the treatment of SLE [117].

Finally, rontalizumab [118], a recombinant humanised monoclonal antibody, was evaluated in a phase II randomised, double-blind, placebo-controlled study (ROSE) but it did not meet the primary endpoint of significant improvement and was withdrawn for further development [119].

Overall, treating refractory SLE patients is challenging because we need to deal with a tremendously heterogeneous disease. It is a reality that biologic therapies are mainly used in clinical scenarios where SLE patients remain resistant to conventional immunosuppressive agents, although it would be highly unlikely that one biologic agent will successfully treat all the disease manifestations, in all patients. Clinicians must beware of the importance of considering each patient as a unique case.

## Why Have Clinical Trials of Biologics in SLE so Often Been Disappointing?

The previous section shows that many biologic agents have not fulfilled their promise in large clinical trials. Rituximab seemed beneficial in open studies [120], epratuzumab [96] gave very promising results in a phase II trial and both abatacept and rontalizumab had good biological reasons to suggest efficacy. Yet all failed to reach the primary endpoint in phase III studies. A number of possible reasons have been proposed to explain this apparent discrepancy and include the following:

1. *Choice of endpoints*—Since SLE is a very complex condition, with potential manifestations in different organs, the concept of response to therapy is more complicated than in rheumatoid arthritis. Most trials have used one of the validated measures of disease activity such as the Systemic Lupus Erythematosus Disease Activity Index (SLEDAI) or British Isles Lupus Assessment Group (BILAG) 2004 index. The indices can be used to derive numerical scores for activity or to define what constitutes a flare of disease. No single index is perfect; all have advantages and disadvantages. The best way to approach this seems to be to design a composite index, incorporating BILAG, SLEDAI and a physician global score. One advantage may be to reduce the apparent responder rate in the ‘placebo’ group. This was done successfully in the belimumab trials [76, 94] using an index where improvement in SLEDAI was the key feature, and less successfully in the epratuzumab trials [96, 97] using an index in which improvement in BILAG was key.

For renal trials, endpoints commonly describe total or partial remission on the basis of measurements such as serum creatinine or urine protein [92]. Where these definitions are very strict, it may make it difficult to recognise truly important clinical improvements. This was exemplified by the differing results when the data from an abatacept trial was analysed in two different ways [103].

2) *Selection of patients*—Many studies exclude patients with active neuropsychiatric or renal disease [76, 92, 94]. This is a problem because these are exactly the types of patients most likely to be refractory to current standard-of-care drugs. One might argue that the best chance of seeing improvements lies in treating patients with the most severe forms of disease.
3) *Treatment of the* ‘*placebo*’ *group*—For ethical reasons, it would not be possible to treat the non-biologic group with placebo alone. Thus, these comparator groups are typically treated with corticosteroids (often in appreciable doses) and immunosuppressants [91, 92]. Since recruitment to trials often rests heavily upon patients with skin and joint activity (which are the most common forms of active lupus, especially when renal and neuropsychiatric activity is excluded from the trial), this level of treatment in the comparator group often leads to a response rate of 40% or higher, meaning that it is very difficult to prove any advantage of the treatment group statistically.
4) *Requirement for large numbers of patients*—It is striking that the BLISS-52 [76] and BLISS-76 [94] belimumab trials, which were almost the only ones to meet their primary endpoint, were also the largest with over 800 patients each. Since SLE is a rare disease, and since many patients respond very well to standard therapy, it is often hard to recruit patients into trials and this problem led to discontinuation of RITUXILUP. Thus, the necessity for finding hundreds of patients over many centres can add to the complexity of such trials and can reduce chances of success.

## Other Clinical Research Challenges in SLE

In this review, we have concentrated on cardiovascular disease, fatigue and development of new therapies as key unmet challenges in patients with SLE. There are other challenges being addressed by researchers in the field.

Development of new biomarkers is important in order to follow the course of the disease and make sure that activity is treated appropriately. For example, neuropsychiatric symptoms occur in a large proportion of patients with SLE, but are usually not due to active disease and do not require increased immunosuppression [121]. Although a variety of different serological and imaging tests have been suggested as aids to diagnosing which patients have true neuropsychiatric SLE, none has achieved sufficient specificity or sensitivity to be adopted into clinical practice (reviewed by Clark et al. [122]). Equally, no serum or urine biomarkers are sufficiently accurate in the diagnosis of incipient or relapsing lupus nephritis that renal biopsies can be replaced.

Management of pregnancy in SLE is another developing field. A large American study showed that 19% of 385 patients with inactive or stable SLE suffered adverse pregnancy outcomes and risk factors increasing the chance of such outcomes included positive lupus anti-coagulant, higher disease activity and anti-hypertensive use [123].
